# SNP Selection in Genome-Wide Association Studies via Penalized Support Vector Machine with MAX Test

**DOI:** 10.1155/2013/340678

**Published:** 2013-09-24

**Authors:** Jinseog Kim, Insuk Sohn, Dennis (Dong Hwan) Kim, Sin-Ho Jung

**Affiliations:** ^1^Department of Statistics and Information Science, Dongguk University, Gyeongju 780-714, Republic of Korea; ^2^Samsung Cancer Research Institute, Samsung Medical Center, Seoul 137-710, Republic of Korea; ^3^Department of Medical Oncology and Hematology, Princess Margaret Hospital, University of Toronto, Toronto, ON, Canada M5G 2M9; ^4^Department of Biostatistics and Bioinformatics, Duke University, Durham, NC 27710, USA

## Abstract

One of main objectives of a genome-wide association study (GWAS) is to develop a prediction model for a binary clinical outcome using single-nucleotide polymorphisms (SNPs) which can be used for diagnostic and prognostic purposes and for better understanding of the relationship between the disease and SNPs. Penalized support vector machine (SVM) methods have been widely used toward this end. However, since investigators often ignore the genetic models of SNPs, a final model results in a loss of efficiency in prediction of the clinical outcome. In order to overcome this problem, we propose a two-stage method such that the the genetic models of each SNP are identified using the MAX test and then a prediction model is fitted using a penalized SVM method. We apply the proposed method to various penalized SVMs and compare the performance of SVMs using various penalty functions. The results from simulations and real GWAS data analysis show that the proposed method performs better than the prediction methods ignoring the genetic models in terms of prediction power and selectivity.

## 1. Introduction

We consider a genome-wide association study (GWAS) on a complex disease. One of the popular study objectives of such study is to predict a binary clinical outcome, such as benign versus malignant and response versus no response with respect to a specific regimen, based on single-nucleotide polymorphisms (SNPs) data. A fitted prediction model will be used to predict the diagnostic or prognostic outcomes of future patients. Recently, penalization approaches incorporating logistic model or support vector machines have been actively proposed to fit prediction models with binary outcomes. These are well known to achieve both predictive accuracy and variable selection simultaneously.

By introducing shrinkage priors of the normal exponential-gamma (NEG) distribution family, Hoggart et al. [1] suggested a stochastic search method for penalized logistic regression models with SNPs. Ayers and Cordell [2] showed that the NEG priors have better performance than other competing penalized methods using simulations, while it is very computing intensive to produce the results. Wu et al. [3] considered lasso-penalized logistic regression [4] with a large number of SNPs and proposed a cyclic coordinate descent algorithm [5] to implement the computation. Kooperberg et al. [6] removed SNPs that had a Hardy-Weinberg *P* value smaller than 10^−5^ and applied logistic regression models with lasso and Elastic net [7] penalties using a set of SNPs preselected by a cross-validation procedure. On the other hand, Wei et al. [8] proposed selecting SNPs using EigenStrat algorithm [9] and applying the SVM and logistic regression as predictive models. Abraham et al. [10] showed that the two penalized methods, *l*_1_ and Elastic-net SVM, were robust in case/control predictive performance based on simulation studies and real data analyses. These simultaneous analysis methods ignored the genetic models of SNPs [6] or assumed the additive model for all SNPs [6, 8, 10].

The statistical tests such as the Pearson's chi-squared test or the Cochran-Armitage trend test (CATT) are frequently used to test if an SNP is associated with a binary outcome by assuming a specific genetic model. Oftentimes, however, the true genetic model is unknown. We can improve the testing power if we know the true genetic model of an SNP [11]. Toward this end, the test based on the maximum over the three CATT statistics (MAX test) has been presented by several authors [12, 13]. Kim et al. [14] recently proposed a prediction method for time-to-event traits using SNPs and showed that a prediction model based on the best fitting genetic models of SNPs can improve the prediction efficiency. We extend their approach to the prediction of binary outcomes using SVMs.

In this paper, we propose a prediction method combining the MAX test and penalized SVM to predict binary outcome using SNPs. The proposed method consists of two phase procedures: (i) to select candidate prognostic SNPs and identify their genetic models using MAX test, and (ii) to fit a prediction model using the penalized SVM with appropriate scores for the selected SNPs based on their genetic types. We compare the performance of the proposed method using a different penalized SVM method through simulations and a real GWAS data analysis. Each SVM method is combined with MAX test or the general practice ignoring the genetic types of the SNPs.

To facilitate and enable MAX test, we provide the R package called SNPselect in http://datamining.dongguk.ac.kr/Rlib/SNPselect which uses the penalized SVM R package [15] to implement SVM with SCAD, *l*_1_, and Elastic Net penalties.

## 2. Methods

### 2.1. Penalized Support Vector Machine

 Suppose that there are *n* subjects. For the subject *i* ( = 1,…, *n*), we have an input vector *x*_*i*_ ∈ *R* ^*p*^ and a class label *y*_*i*_ ∈ {−1,1}. The SVM [16, 17] is to find the optimal hyperplane which separates data points into two classes with the largest margin.

Wahba et al. [18] and Hastie et al. [19] found that the optimization problem of the SVM can be represented as a penalized optimization problem:

(1) $$\begin{array}{c} \underset{\beta_{0},\beta \;}{\min}\overset{n}{\underset{i=1}{\sum }}{\left[1-y_{i}\left(\beta_{0}+\beta^{T}x_{i}\right)\right]}_{+}+p_{\lambda }\left(\beta \right), \end{array}$$

where [1 − *yf*]_+_ = max⁡(1 − *yf*, 0) is called the hinge loss and *p*_*λ*_ is a penality function with regularization parameter *λ*. The SVM using an *l*_2_-norm, *p*_*λ*_(**β**) = ||**β**||_2_^2^, as a penalty function is called the standard SVM or *l*_2_-SVM.

The *l*_2_-SVM has been successfully applied to classification with high-dimensional data such as gene microarrays and SNPs, but it does not select the variables affecting the response class label. For feature selection with *l*_2_-SVM, Guyon et al. [20] proposed the SVM-REF procedure which combines the recursive feature elimination (RFE) with the *l*_2_-SVM. This procedure consists of a two-step procedure using an external gene selection method.

In order to achieve classification accuracy and feature selection simultaneously, variants of SVM have been proposed by replacing the penalty function in (1) with other types of penalty functions, for example, SVM with 1-norm [21, 22], adaptive lasso [23], or smoothly clipped and absolute deviation (SCAD) [24, 25] penalties. The SVM with 1-norm (or *l*_1_-SVM) adapts the lasso (or *l*_1_) penalty, *p*_*λ*_(**β**) = *λ*||**β**||_1_, originally proposed by Tibshirani [4] as a practical alternative to *l*_2_ penalty. Due to the *l*_1_ penalty, the *l*_1_-SVM automatically selects variables by shrinking the small coefficients of the hyperplane to exactly zero.

One of major drawbacks of the *l*_1_ penalty is that it tends to select only one variable when there are many correlated input variables in data. To overcome this limitation of LASSO, Zou and Hastie [7] proposed the Elastic Net penalty by combineing *l*_1_ and *l*_2_ penalties:

(2) $$\begin{array}{c} p_{\lambda }\left(\beta \right)=\lambda_{1}{||\beta ||}_{1}+\lambda_{2}{||\beta ||}_{2}^{2}. \end{array}$$

The Elastic Net penalty provides variable selection owing to *l*_1_ penalty, while finding highly correlated variables, called grouping effect. Wang et al. [26] applied the Elastic Net penalty to SVM classification problems.

Fan and Li [24] proposed the smoothly clipped absolute deviation (SCAD) penalty given as

(3) $$\begin{array}{c} p_{\lambda }\left(\beta \right)=\overset{p}{\underset{j=1}{\sum }}p_{\lambda }\left(\beta_{j};a\right), \end{array}$$

where

(4) $$\begin{array}{c} p_{\lambda }\left(\beta ;a\right)=\{\begin{array}{cc} \lambda \left|\beta \right| & \text{if}\;\;\left|\beta \right|<\lambda \\ -\frac{{\left|\beta \right|}^{2}-2a\lambda \left|\beta \right|+\lambda^{2}}{2\left(a-1\right)} & \text{if}\;\;\lambda \leq \left|\beta \right|\leq a\lambda \\ \frac{\left(a+1\right)\lambda^{2}}{2} & \text{if}\;\;\left|\beta \right|\geq a\lambda . \end{array} \end{array}$$

Here, *a* (>2) and *λ* (>0) are tuning parameters. Fan and Li [24] showed that the prediction with SCAD penalty is not sensitive to the tuning parameter *a* and recommended to use *a* = 3.7.

The SCAD yields the same behavior as *l*_1_ for small coefficients *β*_*j*_, *j* = 1,…, *p*, but assigns a constant penalty for large coefficients. This property reduces the estimation bias. Fan and Li [24] demonstrate more desirable theoretical properties of the SCAD penalty compared to the *l*_1_ penalty. Later, Zhang et al. [25] proposed the SVM with the SCAD penalty for feature selection.

### 2.2. Genetic Models for SNPs

Let AA, AB, and BB be three possible genotypes where B is a risk allele for a given SNP. We denote the number of B alleles in a genotype by *k*; that is, *k* = 0, 1, or 2 if the genotype is AA, AB, or BB, respectively. For a given SNP, the data from *n* patients are summarized in Table 1. 

Let *p*_*k*_ denote the response probability given a genotype *k* = 0,1, 2. If B is the response allele, the response probability increases as the number of B alleles in the SNP increases; that is, *p*_0_ ≤ *p*_1_ ≤ *p*_2_. In this paper, we will consider three popular genetic models satisfying this assumption:

(i)  *recessive* model: *p*_0_ = *p*_1_ < *p*_2_;
(ii)  *dominant* model: *p*_0_ < *p*_1_ = *p*_2_;
(iii)  *additive* model: *p*_0_ < *p*_1_ = (*p*_0_ + *p*_2_)/2.

### 2.3. Trend Test and MAX Test

 For testing association between an SNP and a clinical outcome in case-control studies, the statistical tests such as the Pearson's chi-squared test or CATT are frequently used when the true genetic model is known. In this case, the CATT is usually more powerful than Pearson's chi-squared test when *p*_0_ ≤ *p*_1_ ≤ *p*_2_ [12]. For a single SNP, borrowing the notations of Table 1, the CATT statistic can be written as

(5) $$\begin{array}{c} T_{c}=\frac{n^{1/2}\sum_{k=0}^{2}c_{k}\left(sr_{k}-rs_{k}\right)}{\sqrt{rs\left\{n\sum_{k=0}^{2}c_{k}^{2}n_{k}-{\left(\sum_{k=0}^{2}x_{k}n_{k}\right)}^{2}\right\}}}, \end{array}$$

where (*c*_0_, *c*_1_, and *c*_2_) is a set of scores assigned to genotypes (*AA*, *AB*, and *BB*) with respect to a specific genotype. The trend test is invariant under a linear transformation with *c*_0_ ≤ *c*_1_ ≤ *c*_2_, so that the typical choice of these scores is *c*_0_ = 0 and *c*_2_ = 1, but *c*_1_ can take a different value according to a specific genetic model. From the results of Sasieni [27] and Zheng et al. [12, 28], the optimal choices of *c*_1_ are 0,1/2 and 1 for the recessive, additive, and dominant models, respectively. Let *p*_*k*_ denote the response probability for genotype group *k* = 0,1, 2. Under the null hypothesis of no association, *H*_0_ : *p*_0_ = *p*_1_ = *p*_2_, *T*_*c*_ approximately follows *N*(0,1) for large *n*.

When the true genetic model is unknown, the test based on multiple CATTs for different genetic models can lead to substantial reduction in statistical power [11] or inflated type I error rate. To address this issue, the test based on the maximum over the three CATT statistics (MAX test) has been proposed by several authors [12, 13]. Let *T*_*R*_, *T*_*A*_, and *T*_*D*_ denote the CATT statistics using the scores for recessive, additive, and dominant models, respectively. Based on the three CATT statistics, the MAX test statistic is defined as

(6) $$\begin{array}{c} T_{\max}=\max\left(\left|T_{R}\right|,\left|T_{A}\right|,\left|T_{D}\right|\right). \end{array}$$

The MAX test has robust properties [29] and is more powerful than the Pearson's chi-squared test [12] when the underlying genetic model is unknown.

Even though one can easily calculate the MAX test statistic from (5) and (6), it is not simple to compute its *P* value. One approach of obtaining the *P* value is based on a Monte-Carlo simulation. Under *H*_0_, Zheng et al. [12] showed that (*T*_*R*_, *T*_*D*_, *T*_*A*_) is asymptotically normal with covariances

(7) cov⁡(TR,TA)=f2(f1+2f0)f2(1−f2)f0(f1+2f2)+f2(f1+2f0),cov⁡(TR,TD)=f0f2f0(1−f0)f2(1−f2),cov⁡(TA,TD)=f0(f1+2f2)f0(1−f0)f0(f1+2f2)+f2(f1+2f0),

where *f*_*k*_ denotes the relative frequency of genotype *k* = 0,1, 2. Thus we can approximate the *P* value of MAX test based on Monte-Carlo samples from multivariate normal distribution with estimated variance-covariance matrix $\hat{\Sigma }$ which is obtained by replacing *f*_*k*_ in the above covariances with ${\hat{f}}_{k}=r_{k}/n_{k}$ for *k* = 0,1, 2  (*f*_0_ + *f*_1_ + *f*_2_ = 1).

There have been some studies on variants of MAX test for binary clinical outcomes. Zheng et al. [12] developed a robust ranking method, called MAX-rank test. Conneely et al. [30] proposed an efficient *P* value computation method that is shown to be more accurate than that using permutations by adjusting for correlated test statistics. Li et al. [31] proposed the P-rank test approximating the *P* value for the MAX test with or without covariate adjustment. Li et al. [32] compared the performance of the MAX-rank and P-rank tests. For more detailed discussions on MAX test, see [11] or [32].

### 2.4. Classification via SVM with MAX Test

For patient *i* = 1,…, *n*, let *y*_*i*_ denote the binary clinical outcome taking 1 if responded or −1 if not responded and (*k*_*i*1_,…, *k*_*im*_) the encoded data on *m* SNPs, that is, *k*_*ij*_ = 0,1, 2, the number of the risk allele for SNP *j* ( = 1,…, *m*). To build a classification model with this data set, we propose a method combining a penalized SVM and the MAX test. Our method consists of two-phase procedures: (i) prescreening SNPs and identifying the genetic models for the selected SNPs using the MAX test and (ii) applying the penalized SVM to fit a classification model. Our method can be summarized as follows.

(1) Read in the clinical outcomes (*y*_1_,…, *y*_*n*_) and SNP data {(*k*_*i*1_,…, *k*_*im*_), *i* = 1,…, *n*}.
(2) For SNP *j* ( = 1,…, *m*),
  (a) using the original data, calculate test statistics (*T*_*j*,*R*_, *T*_*j*,*A*_, *T*_*j*,*D*_) and their two-sided *P* values (*p*_*j*,*R*_, *p*_*j*,*A*_, *p*_*j*,*D*_) and MAX test statistic *T*_*j*,max⁡_ = max⁡(|*T*_*j*,*R*_ | , |*T*_*j*,*A*_ | , |*T*_*j*,*D*_|).
  (b) compute the approximate *P* value of MAX test by Monte-Carlo simulation:
    (i) estimate the variance-covarince matrix ${\hat{\Sigma }}_{j}$;
    (ii) generate (*t*_*j*,*R*_^(*b*)^, *t*_*j*,*A*_^(*b*)^, *t*_*j*,*D*_^(*b*)^) from $N(0,{\hat{\Sigma }}_{j})$ for *b* = 1,…, *B* (=100,000, say);
    (iii) approximate the *P* value for MAX test by
      (8) $$\begin{array}{c} p_{j}=B^{-1}\overset{B}{\underset{b=1}{\sum }}I\left(t_{j,\max}^{\left(b\right)}\geq T_{j,\max}\right), \end{array}$$
     where  *t*_*j*,max⁡_^(*b*)^ = max⁡(|*t*_*j*,*R*_^(*b*)^|, |*t*_*j*,*A*_^(*b*)^|, |*t*_*j*,*D*_^(*b*)^|).
(3) SNP screening: select *J* (≪*m*) SNPs with *p*_*j*_ < *α* for a prespecified *α* value, such as 0.01.
(4) For SNP *j*, identify the genetic model by the smallest *P* value among *p*_*j*,*R*_, *p*_*j*,*A*_, and *p*_*j*,*D*_.
(5) Assign covariate values (*z*_*i*1_,…, *z*_*iJ*_) using the score corresponding to the identified genetic model.
(6) Standardize the covariates; that is,
  (9) $$\begin{array}{c} z_{ij}^{\prime }=\frac{z_{ij}-{\overset{-}{z}}_{j}}{s_{j}}, \end{array}$$
 where ${\overset{-}{z}}_{j}=n^{-1}\sum_{i=1}^{n}z_{ij}$ and $s_{j}^{2}=n^{-1}\sum_{i=1}^{n}{\left(z_{ij}-{\overset{-}{z}}_{j}\right)}^{2}$.
(7) Apply the penalized SVM to the response data (*y*_1_,…, *y*_*n*_) and the standardized covariates {(*z*_*i*1_′,…, *z*_*iJ*_′), *i* = 1,…, *n*}.

## 3. Results

### 3.1. Simulation Studies

At first, we generate IID *N*(0,1) random variables *ϵ*_*i*1_,…, *ϵ*_*im*_ and, for *ρ* ∈ (0,1), set

(10) $$\begin{array}{c} x_{ij}=\{\begin{array}{cc} \epsilon_{ij}, & j=1\\ \rho x_{i,j-1}+\sqrt{1-\rho^{2}}\epsilon_{ij}, & j=2,\ldots ,m. \end{array} \end{array}$$

Note that *x*_*i*1_,…, *x*_*im*_ have an AR(1) correlation structure with autocorrelation coefficient *ρ* as in [14]. Correlated SNP data are generated by

(11) $$\begin{array}{c} z_{ij}=\{\begin{array}{cc} 0, & x_{ij}<u_{f_{0}}\\ 1, & u_{f_{0}}\leq x_{ij}<u_{\left(f_{0}+f_{1}\right)}\\ 2, & \text{otherwise}, \end{array} \end{array}$$

where *u*_*q*_ denotes the *q*th quantile of the standard normal distribution. The binary clinical outcome of patient *i* is generated using response probability *p*_*i*_ which is related to the covariates by

(12) $$\begin{array}{c} \text{logit}\left(p_{i}\right)=\overset{m}{\underset{j=1}{\sum }}\beta_{j}z_{ij}. \end{array}$$

To consider the cases of uncorrelated or moderately correlated SNPs in our experiment, we set *ρ* = 0 or 0.3. We generate *m* = 1000 encoded SNPs with (*f*_0_, *f*_1_) = (1/4,1/2) for *j* = 1,…, 6 and (*f*_0_, *f*_1_) = (1/3,1/3) for *j* = 7,…, 1000. SNPs 1 and 2 have recessive models; SNPs 3 and 4 have dominant models, and SNPs 5 and 6 have additive models, the regression coefficients for these six prognostic SNPs are set at *β*_1_ = *β*_2_ = *β*_3_ = *β*_4_ = *β*_5_ = *β*_6_ = 0.8. According to the above data generation scheme, we have generated simulation data sets of size 200, and each data set is partitioned into 2/3 training set and 1/3 test set. For a classification model fitting, the SVM with one of the three penalty functions, SCAD (SCAD-SVM), *l*_1_ (*l*_1_-SVM), and Elastic Net (Enet-SVM), is applied to the SNPs selected using *α* = 0.01. To choose a final classification model, we use 5-fold cross-validation for selecting the tuning parameters. One of the standard practice in the classification model fitting using SNP data will be assuming an equal genetic model for all SNPs. In order to evaluate the performance of the model fitting methods combined with the MAX test, we also have fitted a classification model by assuming one genetic model for all SNPs.

For each model fitting method, we calculate three performance measures such as the number of the selected SNPs, the number of the selected prognostic SNPs by the penalized SVM, and the misclassification error. Here, the selected SNPs are selected by penalized SVM among SNPs after a prescreening step, and the selected prognostic SNPs are the prognostic ones included in the selected SNPs. The misclassification errors are estimated using test data set; that is,

(13) $$\begin{array}{c} \frac{1}{n}\overset{n}{\underset{i=1}{\sum }}I\left(y_{i}\neq \mathrm{sign}\hat{f}\left(z_{i}\right)\right), \end{array}$$

where *I*(*x*) is an indicator function, $\hat{f}(z)={\hat{\beta }}_{1}z_{1}^{\prime }+\cdots +{\hat{\beta }}_{J}z_{J}^{\prime }$ denote the predicted response score predicted for the test set, and *z*_*j*_′s are standardized covariates in the test set using the means and standard errors calculated from the training set. In order to assess the variability of the experiments, we replicate the whole process 100 times.  Table 2 summarizes the three averaged performance measures from our simulations.

When comparing the number of selected SNPs in Table 2, we observe that Enet-SVM tends to select more SNPs but SCAD-SVM selects lower SNPs except for the case of *ρ* = 0.3 and dominant model. In view of different genetic models, the proposed method selects more SNPs when applying *l*_1_-SVM or Enet-SVM. However, the combination of proposed method and SCAD-SVM selects much less SNPs than other combinations. Comparing the numbers of prognostic SNPs, Enet-SVM or *l*_1_-SVM performs better than SCAD-SVM and assuming the proposed method or additive model has good selectivities of the true prognostic SNPs. In results with correlated SNPs (*ρ* = 0.3), Enet-SVM and *l*_1_-SVM with the proposed method result in better selectivities for true prognostic SNPs than those with the additive model. However, the proposed methods can be the worst when SCAD-SVM is used for uncorrelated SNP data. We also compare the misclassification errors. Even if there are a little differences between Enet-SVM and *l*_1_-SVM, Enet-SVM performs better than other penalized methods. SCAD-SVM produces the worst misclassification errors for all cases. We also find that the proposed method has the lowest misclassification errors whatever the penalized SVM method used except the case of applying SCAD-SVM for *ρ* = 0.3. Based on the discussions on the simulation results so far, the proposed method combined with Enet-SVM or *l*_1_-SVM could improve the selectivity for true prognostic SNPs and the ablility of precdiction than other methods using a prefixed genetic model.

### 3.2. Real Data Analysis Example

Kim et al. [33] performed a GWAS using Affymetic Genome-wide Human SNP Arrays 6.0 (San Diego, CA, USA) on 190 patients with chronic myelogenous leukemia (CML). After excluding the SNPs with one missing case and those with the same genotype for all 190 patients, we use 330,353 autosomal SNPs in the further data analysis. The clinical endpoint is the achievement of major molecular response by 18 months to an induction chemotherapy. BCR/ABL transcript levels were measured to determine molecular response to imatinib therapy as described before by Kim et al. [34] and presented using the international scale. Major molecular response (MMR) was defined as <0.1% of the BCR/ABL fusion gene transcript level on an international scale by quantitative PCR. Among the 190 patients, 115 responded.

We randomly partition the CML data into 126 training samples and 64 test samples and then calculate the predictive performance measures for the methods over 100 random partitions.  Table 3 summarizes the number of selected SNPs and the mean misclassification errors with their standard errors in parentheses over 100 random partitions. Similar to the simulation results, *l*_1_-SVM and Enet-SVM using the MAX test slightly increase the number of selections, but produce lower misclassification errorr. Among the three penalized methods, Enet-SVM selects the largest number of SNPs but has the lowest misclassification error regardless of the use of the MAX test. However, SCAD-SVM selects the lowest SNPs, while it has poor prediction performances for any assumption for genetic models, which is the same observation in the simulation results.

Table 4 shows the list of 51 SNPs selected commonly by three penalized methods from 126 training samples of one of 100 random partitions. TGFBR1 gene (rs420549, located in 3′UTR region) among 51 SNPs, transforming growth factor beta receptor 1, interacts with TGF beta 1 [35, 36] and TGF beta receptor 2 [37, 38] and is located in 9q22. TGF beta is playing an important role of maintaining the growth and differentiation balance of hematopoietic cells [39, 40] and is known to have bidirectional properties of tumor suppressing and promoting function [41]. TGF-*β*-FOXO signaling pathway is involved in the maintenance of leukemia-initiating cells in CML, contributing to intrinsic resistance of CML LSCs to tyrosine kinase inhibitor [42, 43]. Accordingly, intrinsic trait of receptor affinity on TGF-*β* might contribute to different sensitivities to TGF-*β*; thus, it is potentially explainable that the response to imatinib therapy is dependent on the TGFBR1 genotype.

## 4. Conclusions

Although the penalized methods have been considered as successful ones for prediction in GWAS, they are still subject to high misclassification error by ignoring the genetic models of prognostic SNPs. In this paper, we proposed a two-phase procedure: (i) carrying out the MAX test for screening out noncandidate SNPs and identifying the genetic models of the selected SNPs at the first stage and then (ii) applying a penalized SVM to the selected SNPs for fitting a classification model at the second stage. We have compared the performances of the proposed method with the conventional methods ignoring the genetic type of prognostic SNPs through simulations and real data example. In the simulations, we observed that Enet-SVM and *l*_1_-SVM select more SNPs but have higher selectivities for true prognostic SNPs and lower misclassification errors among the three penalized SVM methods. Combining the proposed method which selects candidate SNPs and estimates their genetic models, we observed that the penalized SVMs except for SCAD-SVM could improve the performances in terms of the selection of the true prognostic SNPs and misclassification errors. Furthermore, the differences of misclassification errors among the three methods with the proposed method become much smaller. Hence, whichever a penalized SVM for model fitting we use, combining it with the MAX test to identify the genetic models of candidate prognostic SNPs could help to improve its performances. We made similar observations from a real data example. Even so, the selection of candidate SNPs could vary according to the choice of a prespecified *α*; thus, the prescreening by the MAX test could not select a part of true prognostic SNPs. We will consider this point in future work.

## Figures and Tables

**Table 1:** Genotype frequencies.

	AA	AB	BB	Total
Response	*r*_0_	*r*_1_	*r*_2_	*r*
No response	*s*_0_	*s*_1_	*s*_2_	*s*

Total	*n*_0_	*n*_1_	*n*_2_	*n*

**Table 2:** The result of simulations with 100 replications: selected SNPs and prognostic SNPs indicate the averaged numbers of the selected SNPs and the selected prognostic SNPs, respectively, in the fitted models; standard error is reported in the parentheses.

*ρ*	Genetic model	Selected SNPs			Prognostic SNPs			Misclassification error		
		*l*_1_	Enet	SCAD	*l*_1_	Enet	SCAD	*l*_1_	Enet	SCAD
0	Proposed	43.10	48.66	20.31	5.11	5.46	3.31	0.1766	0.1567	0.2736
		(0.54)	(0.70)	(0.65)	(0.09)	(0.07)	(0.14)	(0.0048)	(0.0054)	(0.0062)
	Recessive	40.50	41.38	25.28	4.62	4.74	3.66	0.2408	0.2518	0.2912
		(0.56)	(1.11)	(0.66)	(0.10)	(0.10)	(0.14)	(0.0054)	(0.0065)	(0.0047)
	Additive	42.71	45.58	24.12	5.23	5.35	4.07	0.2161	0.2118	0.3272
		(0.49)	(0.87)	(0.91)	(0.08)	(0.08)	(0.18)	(0.0048)	(0.0064)	(0.0076)
	Dominant	41.35	43.46	23.99	4.70	4.86	3.38	0.2457	0.2347	0.2995
		(0.58)	(0.98)	(0.70)	(0.10)	(0.09)	(0.12)	(0.0056)	(0.0063)	(0.0042)

0.3	Proposed	42.92	45.68	19.56	5.12	5.20	3.40	0.1690	0.1541	0.2833
		(0.50)	(0.80)	(0.48)	(0.08)	(0.08)	(0.10)	(0.0049)	(0.0047)	(0.0060)
	Recessive	39.49	41.09	27.23	4.34	4.47	3.03	0.2383	0.2368	0.2741
		(0.58)	(0.87)	(0.59)	(0.10)	(0.11)	(0.03)	(0.0057)	(0.0057)	(0.0019)
	Additive	42.07	43.90	21.89	5.06	5.04	3.74	0.2126	0.2074	0.3338
		(0.52)	(0.96)	(0.67)	(0.08)	(0.08)	(0.08)	(0.0052)	(0.0057)	(0.0056)
	Dominant	39.97	38.56	24.97	4.56	4.29	2.04	0.2502	0.2338	0.2607
		(0.62)	(1.03)	(0.53)	(0.10)	(0.11)	(0.03)	(0.0065)	(0.0059)	(0.0039)

**Table 3:** The results of CML data: number of selected SNPs and misclassification error are calculated on average over 100 random partitions; standard error is reported in the parentheses.

Genetic model	Average number of selected SNPs			Misclassification error		
	*l*_1_-SVM	Enet-SVM	SCAD-SVM	*l*_1_-SVM	Enet-SVM	SCAD-SVM
Proposed	70.38	99.80	55.90	0.0737	0.0590	0.1098
	(1.29)	(4.10)	(0.52)	(0.0036)	(0.0062)	(0.0013)
Recessive	55.24	120.46	27.82	0.1184	0.0562	0.2003
	(1.19)	(4.73)	(2.33)	(0.0048)	(0.0051)	(0.0044)
Additive	66.32	120.76	43.50	0.1063	0.0667	0.1530
	(1.12)	(5.00)	(0.27)	(0.0051)	(0.0061)	(0.0026)
Dominant	51.90	91.92	50.90	0.1013	0.0702	0.1663
	(0.89)	(4.81)	(1.30)	(0.0062)	(0.0069)	(0.0044)

**Table 4:** List of SNPs selected commonly by three penalized methods.

RS ID	Genetic model	*P* value	RS ID	Genetic model	*P* value	RS ID	Genetic model	*P* value
rs3750551	D	0.000510	rs9289221	R	0.000160	rs6621316	A	0.000890
rs3886721	A	0.000040	rs16972014	A	0.000170	rs9890262	R	0.000210
rs2938451	A	0.000000	rs3013492	R	0.000760	rs6779769	A	0.000510
rs6429646	R	0.000050	rs7095688	A	0.000920	rs9502826	D	0.000690
rs6426870	R	0.000230	rs1439691	R	0.000100	rs9896683	R	0.000850
rs4784924	R	0.000100	rs7123207	R	0.000490	rs12907966	D	0.000220
rs8075266	R	0.000190	rs16830058	A	0.000830	rs5979009	D	0.000150
rs4851920	R	0.000130	rs10484180	R	0.000930	rs17157980	D	0.000730
rs9809817	R	0.000190	rs1952096	A	0.000250	rs2865510	R	0.000160
rs342735	A	0.000180	rs2842068	D	0.000600	rs12457620	D	0.000810
rs17066311	D	0.000790	rs420549	D	0.000440	rs4510937	R	0.000390
rs6627852	A	0.000470	rs16822723	A	0.000590	rs8073928	R	0.000510
rs11841074	D	0.000130	rs2492664	A	0.000270	rs10409991	R	0.000290
rs9447907	R	0.000650	rs2029866	R	0.000730	rs1871332	A	0.000150
rs16873423	D	0.000360	rs764515	A	0.000030	rs1264547	D	0.000670
rs315025	A	0.000390	rs11197596	A	0.000240	rs2016016	A	0.000360
rs2355615	A	0.000130	rs9344734	D	0.000690	rs6605081	R	0.000150
